# On *μ*-*β*-Lindelöf sets in generalized topological spaces

**DOI:** 10.1016/j.heliyon.2023.e13597

**Published:** 2023-02-09

**Authors:** Mohammad S. Sarsak

**Affiliations:** Department of Mathematics, Faculty of Science, The Hashemite University, P.O. Box 330127, Zarqa 13133, Jordan

**Keywords:** Generalized topology, *μ*-open, *μ*-*β*-open, $\omega_{\mu }$-*β*-open, *μ*-*β*-Lindelöf set, *μ*-*β*-Lindelöf space

## Abstract

We introduce and study *μ*-*β*-Lindelöf sets in generalized topological spaces (GTSs) as a subclass of both *μ*-semi-Lindelöf sets and strongly *μ*-Lindelöf sets. We also introduce and study a new type of generalized open sets in GTSs, called $\omega_{\mu }$-*β*-open sets, and apply them to obtain more properties of *μ*-*β*-Lindelöf sets.

## 1 Introduction

In generalized topological spaces (GTSs), Lindelöf properties via weak forms and strong forms of open sets have been of special interests to several mathematicians. The primary purpose of this article is to introduce and study *μ*-*β*-Lindelöf sets in GTSs; several properties and mapping properties of *μ*-*β*-Lindelöf sets are studied extensively.

A generalized topology (GT) [1] *μ* on a non-empty set *X* is a collection of subsets of *X* such that ∅∈μ and *μ* is closed under non-empty arbitrary unions. For a GT *μ* on *X*, the pair (X,μ) will be called a GTS, elements of *μ* will be called *μ*-open sets, and a subset *A* of (X,μ) will be called *μ*-closed if X﹨A is *μ*-open. A space (X,μ) will always mean a GTS. If *A* is a subset of a space (X,μ), then the *μ*-closure of *A* [2], $c_{\mu }\left(A\right)$, is the intersection of all *μ*-closed sets containing *A*, and the *μ*-interior of *A* [2], $i_{\mu }\left(A\right)$, is the union of all *μ*-open sets contained in *A*. A GT *μ* on *X* is called a strong GT [3] if X∈μ. A space (X,μ) is called a *μ*-space [4] if X∈μ, that is *μ* is a strong GT. Strong GTs were introduced by Lugojan [5] (resp. Mashhour et al. [6]) under the name generalized topologies (resp. supra-topologies).

If (X,τ) is a topological space and A⊂X, then $\bar{A}$ and Int *A* will stand, respectively, for the closure of *A* in *X* and the interior of *A* in *X*. A subset *A* of a topological space (X,τ) is called semi-open [7] (resp. preopen [8], *β*-open [9] or called semi-preopen [10], *α*-open [11] if $A\subset \bar{\mathrm{Int}\;A}$ (resp. $A\subset \mathrm{Int}\;\bar{A}$, $A\subset \bar{\mathrm{Int}\;\bar{A}}$, $A\subset \mathrm{Int}\;\bar{\mathrm{Int}\;A}$). The families of semi-open (resp. preopen, *β*-open, *α*-open) subsets of a topological space (X,τ) will be denoted by SO(X) (resp. PO(X), β(X), α(X)). It is known that if μ∈{SO(X),PO(X),β(X),α(X)}, then *μ* is a strong GT on *X*.

Throughout this article, the set-theoretic framework is ZFC, that is the Zermelo-Fraenkel system of axioms (ZF) together with the Axiom of Choice (AC); for a set *A*, |A| stands for the cardinality of *A*.

## 2 Preliminaries

We recall the following definitions and facts for their importance in the material of our article.

Definition 2.1 [2] Let *A* be a subset of a space (X,μ). Then *A* is called

(i) *μ*-semi-open if $A\subset c_{\mu }\left(i_{\mu }\left(A\right)\right)$;

(ii) *μ*-preopen if $A\subset i_{\mu }\left(c_{\mu }\left(A\right)\right)$;

(iii) *μ*-*β*-open if $A\subset c_{\mu }\left(i_{\mu }\left(c_{\mu }\left(A\right)\right)\right)$;

(iv) *μ*-*α*-open if $A\subset i_{\mu }\left(c_{\mu }\left(i_{\mu }\left(A\right)\right)\right)$.

For a space (X,μ), we will denote the class of *μ*-semi-open sets by $\sigma_{\mu }$ or *σ*, the class of *μ*-preopen sets by $\pi_{\mu }$ or *π*, the class of *μ*-*β*-open sets by $\beta_{\mu }$ or *β*, and the class of *μ*-*α*-open sets by $\alpha_{\mu }$ or *α*. It was pointed out in [2] that for a space (X,μ), each of the families *σ*, *π*, *α*, and *β* is a GT. However, it is easy to see that for a space (X,μ), each of the families *σ* and *β* is a strong GT, while *π* and *α* need not.

Proposition 2.2 *[2]* *Let* (X,μ) *be a space. Then*

*(i)* μ⊂α=σ∩π *;*

*(ii)* σ∪π⊂β *;*

*(iii)* $\pi_{\sigma }=\pi_{\beta }=\beta$ *;*

*(iv)* $\beta_{\sigma }=\beta_{\alpha }=\beta_{\beta }=\beta$ *.*

Definition 2.3 [12] (i) A subset *A* of a *μ*-space (X,μ) is called *μ*-Lindelöf if any cover of *A* by *μ*-open subsets of *X* has a countable subcover.

(ii) A *μ*-space (X,μ) is called *μ*-Lindelöf if any cover of *X* by *μ*-open sets has a countable subcover.

Definition 2.4 (i) A subset *A* of a topological space (X,τ) is called strongly Lindelöf [13] relative to (X,τ) if any cover of *A* by preopen subsets of *X* has a countable subcover.

(ii) A topological space (X,τ) is called strongly Lindelöf [14] if any cover of *X* by preopen subsets of *X* has a countable subcover.

Definition 2.5 [15] (i) A subset *A* of a *μ*-space (X,μ) is called strongly *μ*-Lindelöf if any cover of *A* by *μ*-preopen subsets of *X* has a countable subcover, that is *A* is $\pi_{\mu }$-Lindelöf.

(ii) A *μ*-space (X,μ) is called strongly *μ*-Lindelöf if any cover of *X* by *μ*-preopen sets has a countable subcover, that is $\left(X,\pi_{\mu }\right)$ is $\pi_{\mu }$-Lindelöf.

Definition 2.6 (i) A subset *A* of a topological space (X,τ) is called semi-Lindelöf in *X* [16] if any cover of *A* by semi-open subsets of *X* has a countable subcover.

(ii) A topological space (X,τ) is called semi-Lindelöf [17] if any cover of *X* by semi-open subsets of *X* has a countable subcover.

Definition 2.7 (i) A subset *A* of a space (X,μ) is called *μ*-semi-Lindelöf relative to *X* [18] if any cover of *A* by *μ*-semi-open subsets of *X* has a countable subcover, or equivalently [19], *A* is $\sigma_{\mu }$-Lindelöf. We will say *A* is *μ*-semi Lindelöf to mean *A* is *μ*-semi Lindelöf relative to *X*.

(ii) A space (X,μ) is called *μ*-semi-Lindelöf [18] if any cover of *X* by *μ*-semi-open sets has a countable subcover, or equivalently [19], $\left(X,\sigma_{\mu }\right)$ is $\sigma_{\mu }$-Lindelöf.

Proposition 2.8 *Let A be a subset of a topological space X. Then*

*(i)* *[7]* *A is semi-open if and only if there exists an open set U such that* $U\subset A\subset \bar{U}$ *.*

*(ii)* *[20]* *A is preopen if and only if* A=U∩D *, where U is open and D is dense.*

*(iii)* *[21]* *A is β-open if and only if* A=S∩D *, where S is semi-open and D is dense.*

## 3 *μ*-*β*-Lindelöf sets

This section is mainly devoted to introducing and studying *μ*-*β*-Lindelöf sets in GTSs.

Definition 3.1 [15] (i) A subset *A* of a topological space (X,τ) is called *β*-Lindelöf relative to (X,τ) if any cover of *A* by *β*-open subsets of *X* has a countable subcover.

(ii) A topological space (X,τ) is called *β*-Lindelöf if any cover of *X* by *β*-open subsets of *X* has a countable subcover.

Definition 3.2 (i) A subset *A* of a space (X,μ) is called *μ*-*β*-Lindelöf if any cover of *A* by *μ*-*β*-open subsets of *X* has a countable subcover, that is *A* is $\beta_{\mu }$-Lindelöf.

(ii) A space (X,μ) is called *μ*-*β*-Lindelöf if any cover of *X* by *μ*-*β*-open sets has a countable subcover, that is $\left(X,\beta_{\mu }\right)$ is $\beta_{\mu }$-Lindelöf.

Remark 3.3 It is easy to see that the countable union of *μ*-*β*-Lindelöf subsets of a space (X,μ) is *μ*-*β*-Lindelöf.

The following two remarks follow from Proposition 2.2(iv).

Remark 3.4 Let *A* be a subset of a space (X,μ). Then it is clear that

(i) *A* is *μ*-*β*-Lindelöf if and only if *A* is $\sigma_{\mu }$-*β*-Lindelöf;

(ii) *A* is *μ*-*β*-Lindelöf if and only if *A* is $\alpha_{\mu }$-*β*-Lindelöf;

(iii) *A* is *μ*-*β*-Lindelöf if and only if *A* is $\beta_{\mu }$-*β*-Lindelöf.

Remark 3.5 If *A* is a subset of a topological space (X,τ) and μ∈{τ,SO(X),α(X),β(X)}, then

A is μ-β-Lindelöf⇔A is β-Lindelöf relative to (X,τ).

In particular,

(X,μ) is μ-β-Lindelöf⇔(X,τ) is β-Lindelöf.

The next remark follows from Proposition 2.2(iii).

Remark 3.6 Let *A* be a subset of a space (X,μ). Then it is clear that

(i) *A* is *μ*-*β*-Lindelöf if and only if *A* is strongly $\sigma_{\mu }$-Lindelöf;

(ii) *A* is *μ*-*β*-Lindelöf if and only if *A* is strongly $\beta_{\mu }$-Lindelöf.

Now, we will study the relationships between *μ*-*β*-Lindelöf sets and some other related types.

Remark 3.7 Clearly, if a subset *A* of a space (X,μ) is *μ*-*β*-Lindelöf, then *A* is *μ*-semi-Lindelöf; in particular, if a space (X,μ) is *μ*-*β*-Lindelöf, then (X,μ) is *μ*-semi-Lindelöf. However, the converse need not be true even for topological spaces as it can be easily seen from each of the following two examples.

Example 3.8 Let *X* be an uncountable set equipped with the co-countable topology. Then it follows from Proposition 2.8(i) that every semi-open subset of *X* is open, but *X* is Lindelöf, so *X* is semi-Lindelöf. On the other hand, since the dense subsets of *X* are the uncountable subsets of *X*, it follows from Proposition 2.8(ii) that the non-empty preopen subsets of *X* are the uncountable subsets of *X*. As *X* is uncountable, there exists a disjoint family $U=\left\{U_{\alpha }:\alpha \in \Lambda \right\}$ such that X=⋃U, and $\left|U_{\alpha }\right|=\left|\Lambda \right|=\left|X\right|$ for each α∈Λ. Therefore, U is a cover of *X* by preopen sets and U has no countable subcover, that is *X* is not strongly Lindelöf, and thus, *X* is not *β*-Lindelöf.

Example 3.9 Let *X* be an uncountable set equipped with the indiscrete topology. Then it follows from Proposition 2.8(i) that every semi-open subset of *X* is open, but *X* is Lindelöf, so *X* is semi-Lindelöf. On the other hand, since every singleton subset of *X* is preopen, it is clear that *X* is not strongly Lindelöf, and thus, *X* is not *β*-Lindelöf.

Remark 3.10 Clearly, if a subset *A* of a *μ*-space (X,μ) is *μ*-*β*-Lindelöf, then *A* is strongly *μ*-Lindelöf, in particular, if a *μ*-space (X,μ) is *μ*-*β*-Lindelöf, then (X,μ) is strongly *μ*-Lindelöf. However, the converse need not true even for topological spaces as it can be easily seen from the following example.

Example 3.11 Let *X* be an uncountable discrete space, p∉X, and $X^{⁎}=X\cup \left\{p\right\}$. Topologize $X^{⁎}$ as follows: basic open neighborhoods of *X* are unchanged and a basic open neighborhood of *p* has the form {p}∪(X﹨A), where *A* is a countable subset of *X*. Then it is easy to see that $X^{⁎}$ is Lindelöf. Since the only dense subsets of $X^{⁎}$ are *X* and $X^{⁎}$ itself, it follows from Proposition 2.8(ii) that the preopen subsets of $X^{⁎}$ are the open sets, but $X^{⁎}$ is Lindelöf, so $X^{⁎}$ is strongly Lindelöf. On the other hand, we observe that any set of the form {p}∪A, where *A* is an uncountable subset of *X*, is semi-open in $X^{⁎}$. As *X* is uncountable, there exists a disjoint family $U=\left\{U_{\alpha }:\alpha \in \Lambda \right\}$ such that X=⋃U, and $\left|U_{\alpha }\right|=\left|\Lambda \right|=\left|X\right|$ for each α∈Λ. Therefore, $V=\left\{\left\{p\right\}\cup U_{\alpha }:\alpha \in \Lambda \right\}$ is a cover of $X^{⁎}$ by semi-open sets and V has no countable subcover, that is $X^{⁎}$ is not semi-Lindelöf, and thus, $X^{⁎}$ is not *β*-Lindelöf.

Recall that a subset *A* of a space (X,μ) is called *μ*-*β*-closed [22] if X﹨A is *μ*-*β*-open. The following proposition tells that a *μ*-*β*-closed subset of a *μ*-*β*-Lindelöf space is *μ*-*β*-Lindelöf; the straightforward proof is omitted.

Proposition 3.12 *Let A be a μ-β-Lindelöf subset of a space* (X,μ) *and B be a μ-β-closed subset of X. Then* A∩B *is μ-β-Lindelöf. In particular, a μ-β-closed subset A of a μ-β-Lindelöf space* (X,μ) *is μ-β-Lindelöf.*

Proposition 3.13 *If every proper μ-β-closed subset of a space* (X,μ) *is μ-β-Lindelöf, then* (X,μ) *is μ-β-Lindelöf.*

Proof Let $U=\left\{U_{\alpha }:\alpha \in \Lambda \right\}$ be a cover of *X* by *μ*-*β*-open subsets of *X*. Choose $\alpha_{0}\in \Lambda$ such that $U_{\alpha_{0}}\neq \emptyset$. Then $X﹨U_{\alpha_{0}}$ is a proper *μ*-*β*-closed subset of *X*, thus by assumption, $X﹨U_{\alpha_{0}}$ is *μ*-*β*-Lindelöf, so there exist $\alpha_{1},\alpha_{2},\alpha_{3},...\in \Lambda$ such that $X﹨U_{\alpha_{0}}\subset {⋃}_{i=1}^{\infty }U_{\alpha_{i}}$, and thus, $X={⋃}_{i=0}^{\infty }U_{\alpha_{i}}$. Hence, *X* is *μ*-*β*-Lindelöf. □

As for the next issue, we characterize *μ*-*β*-Lindelöf sets in a subspace; to proceed, we recall the following definition.

Definition 3.14 [12] Let *A* be a non-empty subset of a space (X,μ). The generalized subspace topology on *A* is the collection {U∩A:U∈μ}, which will be denoted by $\mu_{A}$. The generalized subspace *A* is the GTS $\left(A,\mu_{A}\right)$.

Proposition 3.15 *Let B be a non-empty subset of a space* (X,μ) *and* A⊂B *. Then A is μ-β-Lindelöf if and only if A is* ${\left(\beta_{\mu }\right)}_{B}$ *-Lindelöf.*

Proof **Necessity.** Suppose that $A=\left\{A_{\alpha }:\alpha \in \Lambda \right\}$ is a cover of *A* by ${\left(\beta_{\mu }\right)}_{B}$-open sets. Then $A_{\alpha }=S_{\alpha }\cap B$, where $S_{\alpha }$ is $\beta_{\mu }$-open for each α∈Λ. Thus $S=\left\{S_{\alpha }:\alpha \in \Lambda \right\}$ is a cover of *A* by *μ*-*β*-open sets, but *A* is *μ*-*β*-Lindelöf, so there exist $\alpha_{1},\alpha_{2},\alpha_{3},...\in \Lambda$ such that $A\subset {⋃}_{i=1}^{\infty }S_{\alpha_{i}}$, and thus, $A\subset {⋃}_{i=1}^{\infty }\left(S_{\alpha_{i}}\cap B\right)={⋃}_{i=1}^{\infty }A_{\alpha_{i}}$. Hence, *A* is ${\left(\beta_{\mu }\right)}_{B}$-Lindelöf.

**Sufficiency.** Suppose that $S=\left\{S_{\alpha }:\alpha \in \Lambda \right\}$ is a cover of *A* by *μ*-*β*-open sets. Then $A=\left\{S_{\alpha }\cap B:\alpha \in \Lambda \right\}$ is a ${\left(\beta_{\mu }\right)}_{B}$-open cover of *A*, but *A* is ${\left(\beta_{\mu }\right)}_{B}$-Lindelöf, so there exist $\alpha_{1},\alpha_{2},\alpha_{3},...\in \Lambda$ such that $A\subset {⋃}_{i=1}^{\infty }\left(S_{\alpha_{i}}\cap B\right)\subset {⋃}_{i=1}^{\infty }S_{\alpha_{i}}$. Hence, *A* is *μ*-*β*-Lindelöf. □

Corollary 3.16 *Let A be a non-empty subset of a space* (X,μ) *. Then A is μ-β-Lindelöf if and only if A is* ${\left(\beta_{\mu }\right)}_{A}$ *-Lindelöf.*

The proof of the next proposition is straightforward, and thus omitted.

Proposition 3.17 *A non-empty subset A of a space* (X,μ) *is μ-β-Lindelöf if and only if for every family* $F=\left\{F_{\alpha }:\alpha \in \Lambda \right\}$ *of μ-β-closed sets having the property that for every non-empty countable subfamily* $F^{i}$ *of* F *,* $\left(⋂F^{i}\right)\cap A\neq \emptyset$ *, then* (⋂F)∩A≠∅ *.*

Now, we will characterize *μ*-*β*-Lindelöf sets using filter bases; to proceed, we recall the following definition.

Definition 3.18 [15] (i) A filter base F on a non-empty set *X* is called a strong filter base on *X* if whenever $F^{i}$ is a non-empty countable subcollection of F, there exists F∈F such that $F\subset ⋂F^{i}$;

(ii) A strong filter base F on a non-empty set *X* is called a maximal strong filter base on *X* if whenever H is a strong filter base on *X* with F⊂H, then F=H.

It was pointed out in [15] that every strong filter base F on a non-empty set *X* is contained in a maximal strong filter base on *X*.

Definition 3.19 A filter base F on a space (X,μ) is said to $\beta_{\mu }$-converge to a point x∈X if for each *μ*-*β*-open subset *U* of *X* such that x∈U, there exists F∈F such that F⊂U. F is said to $\beta_{\mu }$-accumulate at x∈X if U∩F≠∅ for every F∈F and for every *μ*-*β*-open subset *U* of *X* such that x∈U.

Remark 3.20 Let F be a filter base on a space (X,μ) and x∈X. Then it is easy to see that

(i) If F $\beta_{\mu }$-converges to *x*, then F $\beta_{\mu }$-accumulates at *x*;

(ii) If F is a maximal filter base (maximal strong filter base), then F $\beta_{\mu }$-converges to *x* if and only if F $\beta_{\mu }$-accumulates at *x*.

The next proposition meets our goal for characterizing *μ*-*β*-Lindelöf sets using filter bases, the straightforward proof is omitted.

Proposition 3.21 *For a non-empty subset A of a space* (X,μ) *, the following are equivalent:*

*(i) A is μ-β-Lindelöf;*

*(ii) Every maximal strong filter base on X, each of whose members meet A,* $\beta_{\mu }$ *-converges to some point of A;*

*(iii) Every strong filter base on X, each of whose members meet A,* $\beta_{\mu }$ *-accumulates at some point of A.*

At the end of this section, we study a sum property of *μ*-*β*-Lindelöf spaces; to proceed, we recall the following.

Definition 3.22 [12] Let $\left(X_{\alpha },\mu_{\alpha }\right)$ be a GTS for each α∈Λ, where $\left\{X_{\alpha }:\alpha \in \Lambda \right\}$ is a disjoint family of sets. The collection *μ* of subsets of $⋃X_{\alpha }$ is defined as follows:

$$\mu =\left\{U\subset ⋃X_{\alpha }:U\cap X_{\alpha }\in \mu_{\alpha },\forall \;\alpha \in \Lambda \right\}.$$

Remark 3.23 [12] Let $\left(X_{\alpha },\mu_{\alpha }\right)$ be a GTS for each α∈Λ, where $\left\{X_{\alpha }:\alpha \in \Lambda \right\}$ is a disjoint family of sets, and let *μ* be as in Definition 3.22. Then *μ* is a GT on $⋃X_{\alpha }$. The GTS $\left(⋃X_{\alpha },\mu \right)$ will be called the generalized topological sum of $X_{\alpha },\alpha \in \Lambda$, and will be denoted by $⊕X_{\alpha }$.

Remark 3.24 [12] Let $\left(X_{\alpha },\mu_{\alpha }\right)$ be a $\mu_{\alpha }$-space for each α∈Λ, and let $\left(⊕X_{\alpha },\mu \right)$ be the generalized topological sum of $\left(X_{\alpha },\mu_{\alpha }\right)$, α∈Λ. Then it is easy to see that $X_{\alpha }$ is both *μ*-open and *μ*-closed for each α∈Λ.

Proposition 3.25 *Let*$\left(X_{\alpha },\mu_{\alpha }\right)$*be a*$\mu_{\alpha }$*-space for each*α∈Λ*, and let*$\left(⊕X_{\alpha },\mu \right)$*be the generalized topological sum of*$\left(X_{\alpha },\mu_{\alpha }\right),\alpha \in \Lambda$*. Then*$⊕X_{\alpha }$*is μ-β-Lindelöf if and only if*$X_{\alpha }$*is μ-β-Lindelöf for each*α∈Λ*and* Λ *is countable.*

Proof **Necessity.** By Remark 3.24, $X_{\alpha }$ is *μ*-*β*-closed for each α∈Λ. Since $⊕X_{\alpha }$ is *μ*-*β*-Lindelöf, it follows by Proposition 3.12 that $X_{\alpha }$ is *μ*-*β*-Lindelöf for each α∈Λ. Also by Remark 3.24, $X_{\alpha }$ is *μ*-*β*-open for each α∈Λ, thus, $A=\left\{X_{\alpha }:\alpha \in \Lambda \right\}$ is a cover of $⊕X_{\alpha }$ by *μ*-*β*-open sets, but $⊕X_{\alpha }$ is *μ*-*β*-Lindelöf, so, Λ is countable.

**Sufficiency.** Follows from Remark 3.3. □

## 4 $\omega_{\mu }$-*β*-open sets

In this section, we introduce and study a new type of generalized open sets in GTSs, called $\omega_{\mu }$-*β*-open sets. This study will be useful later to obtain further properties of *μ*-*β*-Lindelöf sets in GTSs.

Definition 4.1 Let (X,μ) be a space and *A* be a subset of *X*. Then *A* is called $\omega_{\mu }$-*β*-open if whenever x∈A, there exists a *μ*-*β*-open set $U_{x}$ containing *x* such that $U_{x}﹨A$ is countable. *A* is called $\omega_{\mu }$-*β*-closed if X﹨A is $\omega_{\mu }$-*β*-open. The collection of all $\omega_{\mu }$-*β*-open subsets of *X* will be denoted by $\omega_{\beta_{\mu }}$.

Proposition 4.2 *(i) If A is a μ-β-open subset of a space* (X,μ) *, then A is* $\omega_{\mu }$ *-β-open, that is* $\beta_{\mu }\subset \omega_{\beta_{\mu }}$ *.*

*(ii) If* (X,μ) *is a space, then* $\left(X,\omega_{\beta_{\mu }}\right)$ *is an* $\omega_{\beta_{\mu }}$ *-space.*

*(iii) Let A be a subset of a space* (X,μ) *. Then A is* $\omega_{\mu }$ *-β-open if and only if A is* $\omega_{\omega_{\beta_{\mu }}}$ *-β-open.*

Proof (i) Let *A* be *μ*-*β*-open and x∈A. Then A﹨A=∅ is countable. Thus, *A* is $\omega_{\mu }$-*β*-open.

(ii) Clearly, ∅ is $\omega_{\mu }$-*β*-open, that is $\emptyset \in \omega_{\beta_{\mu }}$. Now let $U_{\alpha }$ be $\omega_{\mu }$-*β*-open for each α∈Λ, and let $x\in {⋃}_{\alpha \in \Lambda }U_{\alpha }$. Then $x\in U_{\alpha_{0}}$ for some $\alpha_{0}\in \Lambda$. Since $U_{\alpha_{0}}$ is $\omega_{\mu }$-*β*-open, there exists a *μ*-*β*-open set $V_{\alpha_{0}}$ containing *x* such that $V_{\alpha_{0}}﹨U_{\alpha_{0}}$ is countable. Thus, $V_{\alpha_{0}}﹨\left({⋃}_{\alpha \in \Lambda }U_{\alpha }\right)$ is countable. Hence, ${⋃}_{\alpha \in \Lambda }U_{\alpha }$ is $\omega_{\mu }$-*β*-open, that is ${⋃}_{\alpha \in \Lambda }U_{\alpha }\in \omega_{\beta_{\mu }}$. Hence, $\left(X,\omega_{\beta_{\mu }}\right)$ is a space. Now since *X* is *μ*-*β*-open, it follows by (i) that *X* is $\omega_{\mu }$-*β*-open, that is $X\in \omega_{\beta_{\mu }}$. Hence, $\left(X,\omega_{\beta_{\mu }}\right)$ is an $\omega_{\beta_{\mu }}$-space.

(iii) Suppose that *A* is $\omega_{\mu }$-*β*-open and let x∈A. Then there exists a *μ*-*β*-open set *U* containing *x* such that U﹨A is countable. By (i), *U* is $\omega_{\mu }$-*β*-open. By Proposition 2.2(iv), *U* is $\omega_{\beta_{\mu }}$-*β*-open. Thus, *A* is $\omega_{\omega_{\beta_{\mu }}}$-*β*-open. Conversely, suppose that *A* is $\omega_{\omega_{\beta_{\mu }}}$-*β*-open and let x∈A. Then there exists an $\omega_{\beta_{\mu }}$-*β*-open set *U* containing *x* such that U﹨A is countable. By Proposition 2.2(iv), *U* is $\omega_{\mu }$-*β*-open. Thus, there exists a *μ*-*β*-open set *V* containing *x* such that V﹨U is countable. Now V﹨A=((V﹨U)﹨A)∪((U∩V)﹨A). As U﹨A and V﹨U are both countable, (V﹨U)﹨A and (U∩V)﹨A are both countable, and thus, V﹨A is countable. Hence, *A* is $\omega_{\mu }$-*β*-open. □

The following example shows that if (X,μ) is a topological space, then $\left(X,\omega_{\beta_{\mu }}\right)$ need not be, in general, a topological space.

Example 4.3 Let (X,μ) be the topological space of Example 3.8, that is *X* is an uncountable set and *μ* is the co-countable topology on *X*. Since the dense subsets of *X* are the uncountable subsets of *X* and since every semi-open subset of *X* is open, it follows from Proposition 2.8(iii) that the non-empty *μ*-*β*-open subsets of *X* are the uncountable subsets of *X*. We will show now that $\omega_{\beta_{\mu }}$ is not a topology on *X*. Let *A* and *B* be any uncountable subsets of *X* such that A∩B is countable and non-empty. Then *A* and *B* are *μ*-*β*-open, and thus $\omega_{\mu }$-*β*-open by Proposition 4.2(i). However, A∩B is not $\omega_{\mu }$-*β*-open because if x∈A∩B, then there is no uncountable subset $U_{x}$ of *X* such that $x\in U_{x}$ and $U_{x}﹨\left(A\cap B\right)$ is countable. Hence, $\omega_{\beta_{\mu }}$ is not a topology on *X*.

Remark 4.4 It is easy to see that if *A* is a countable subset of a space (X,μ), then *A* is $\omega_{\mu }$-*β*-closed. However, the converse need not be true, in general, even for topological spaces as the following example tells.

Example 4.5 Let *X* be an uncountable set equipped with the discrete topology *μ*. Then clearly every subset of *X* is *μ*-*β*-open; thus by Proposition 4.2(i), every subset of *X* is $\omega_{\mu }$-*β*-open. Now let *A* be a subset of *X* such that X﹨A is uncountable. Then X﹨A is $\omega_{\mu }$-*β*-closed which is uncountable.

The next example shows that the converse of Proposition 4.2(i) need not be correct, in general, even for topological spaces.

Example 4.6 Let *X* be an uncountable set and μ={∅,X,A}, where *A* is a non-empty countable subset of *X*. Then by Remark 4.4, X﹨A is $\omega_{\mu }$-*β*-open; however, X﹨A is not *μ*-*β*-open because

$$X﹨A⊄c_{\mu }\left(i_{\mu }\left(c_{\mu }\left(X﹨A\right)\right)\right)=c_{\mu }\left(i_{\mu }\left(X﹨A\right)\right)=c_{\mu }\left(\emptyset \right)=\emptyset .$$

The following proposition characterizes $\omega_{\mu }$-*β*-open sets in terms of *μ*-*β*-open sets and countable sets.

Proposition 4.7 *Let* (X,μ) *be a space and A be a subset of X. Then A is* $\omega_{\mu }$ *-β-open if and only if whenever* x∈A *, there exists a μ-β-open set* $U_{x}$ *containing x and a countable subset* $C_{x}$ *of X such that* $U_{x}﹨C_{x}\subset A$ *.*

Proof **Necessity.** Let *A* be $\omega_{\mu }$-*β*-open and x∈A. Then there exists a *μ*-*β*-open set $U_{x}$ containing *x* such that $U_{x}﹨A$ is countable. Let $C_{x}=U_{x}﹨A$. Then $U_{x}﹨C_{x}\subset A$.

**Sufficiency.** Let x∈A. Then by assumption, there exists a *μ*-*β*-open set $U_{x}$ containing *x* and a countable subset $C_{x}$ of *X* such that $U_{x}﹨C_{x}\subset A$. Thus, $U_{x}﹨A\subset C_{x}$, and therefore, $U_{x}﹨A$ is countable. Hence, *A* is $\omega_{\mu }$-*β*-open. □

Corollary 4.8 *Let* (X,μ) *be a space and A be an* $\omega_{\mu }$ *-β-closed subset of X. Then* A⊂B∪C *for some μ-β-closed subset B of X and some countable subset C of X.*

Proof Let *A* be an $\omega_{\mu }$-*β*-closed subset of *X*. Then X﹨A is $\omega_{\mu }$-*β*-open. If X﹨A=∅, choose B=X and C=∅. If X﹨A≠∅, choose x∈X﹨A. Thus by Proposition 4.7, there exists a *μ*-*β*-open set $U_{x}$ containing *x* and a countable subset $C_{x}$ of *X* such that $U_{x}﹨C_{x}\subset X﹨A$. Therefore, $A\subset \left(X﹨U_{x}\right)\cup C_{x}$. Let $B=X﹨U_{x}$ and $C=C_{x}$. Then *B* is *μ*-*β*-closed, and A⊂B∪C. □

The next proposition describes the shape of an $\omega_{\mu }$-*β*-open *μ*-*β*-Lindelöf set.

Proposition 4.9 *Let A be an* $\omega_{\mu }$ *-β-open subset of a space* (X,μ) *. If A is μ-β-Lindelöf, then* A=B﹨C *for some μ-β-open subset B of X and some countable subset C of X.*

Proof Since *A* is $\omega_{\mu }$-*β*-open, it follows that for each x∈A there exists a *μ*-*β*-open set $U_{x}$ containing *x* such that $U_{x}﹨A$ is countable, but *A* is *μ*-*β*-Lindelöf, so there exist $x_{1},x_{2},x_{3},...\in A$ such that $A\subset {⋃}_{i=1}^{\infty }U_{x_{i}}$. Thus, $A=\left({⋃}_{i=1}^{\infty }U_{x_{i}}\right)﹨\left({⋃}_{i=1}^{\infty }\left(U_{x_{i}}﹨A\right)\right)$. Let $B={⋃}_{i=1}^{\infty }U_{x_{i}}$ and $C={⋃}_{i=1}^{\infty }\left(U_{x_{i}}﹨A\right)$. Then *B* is *μ*-*β*-open and *C* is countable. □

## 5 More properties

The primary purpose of this section is to provide more properties and mapping properties of *μ*-*β*-Lindelöf sets in GTSs.

Proposition 5.1 *Let A be a subset of a space* (X,μ) *. Then A is μ-β-Lindelöf if and only if A is* $\omega_{\beta_{\mu }}$ *-Lindelöf.*

Proof **Necessity.** Suppose that $S=\left\{S_{\alpha }:\alpha \in \Lambda \right\}$ is a cover of *A* by $\omega_{\beta_{\mu }}$-open sets. Then for each x∈A, $x\in S_{\alpha \left(x\right)}$ for some α(x)∈Λ. Thus, there exists a *μ*-*β*-open set $U_{x}$ containing *x* such that $U_{x}﹨S_{\alpha \left(x\right)}$ is countable. Now $A=\left\{U_{x}:x\in A\right\}$ is a cover of *A* by *μ*-*β*-open sets, but *A* is *μ*-*β*-Lindelöf, so there exist $x_{1},x_{2},x_{3},...\in A$ such that $A\subset {⋃}_{i=1}^{\infty }\left(U_{x_{i}}\cap A\right)\subset \left({⋃}_{i=1}^{\infty }\left(\left(U_{x_{i}}﹨S_{\alpha \left(x_{i}\right)}\right)\cap A\right)\right)\cup \left({⋃}_{i=1}^{\infty }S_{\alpha \left(x_{i}\right)}\right)$. Since ${⋃}_{i=1}^{\infty }\left(\left(U_{x_{i}}﹨S_{\alpha \left(x_{i}\right)}\right)\cap A\right)$ is a countable subset of *A*, it is covered by a countable subcollection B of S. Thus, S has $B\cup \left\{S_{\alpha \left(x_{i}\right)}:i\in N\right\}$ as a countable subcover. Hence, *A* is $\omega_{\beta_{\mu }}$-Lindelöf.

**Sufficiency.** Follows from Proposition 4.2(i). □

Proposition 5.2 *Let A be a μ-β-Lindelöf subset of a space* (X,μ) *and B be an* $\omega_{\mu }$ *-β-closed subset of X. Then* A∩B *is μ-β-Lindelöf. In particular, an* $\omega_{\mu }$ *-β-closed subset A of a μ-β-Lindelöf space* (X,μ) *is μ-β-Lindelöf.*

Proof Suppose that $S=\left\{S_{\alpha }:\alpha \in \Lambda \right\}$ is a cover of A∩B by *μ*-*β*-open sets. Then $A=\left\{S_{\alpha }:\alpha \in \Lambda \right\}\cup \left\{X﹨B\right\}$ is a cover of *A* by $\omega_{\mu }$-*β*-open sets, but *A* is *μ*-*β*-Lindelöf, so it follows from Proposition 5.1 that there exist $\alpha_{1},\alpha_{2},\alpha_{3},...\in \Lambda$ such that $A\subset \left({⋃}_{i=1}^{\infty }S_{\alpha_{i}}\right)\cup \left(X﹨B\right)$. Thus, $A\cap B\subset {⋃}_{i=1}^{\infty }\left(S_{\alpha_{i}}\cap B\right)\subset {⋃}_{i=1}^{\infty }S_{\alpha_{i}}$. Hence, A∩B is *μ*-*β*-Lindelöf. □

The next issue is to study several mapping properties of *μ*-*β*-Lindelöf sets in GTSs.

Recall that a function f:(X,μ)→(Y,κ) is called (μ,κ)-continuous [23] if the inverse image of each *κ*-open set is *μ*-open, and called (μ,κ)-closed [23] if the image of every *μ*-closed set is *κ*-closed.

Proposition 5.3 *Let* f:(X,μ)→(Y,κ) *be a* $\left(\omega_{\beta_{\mu }},\beta_{\kappa }\right)$ *-continuous function, where* (X,μ) *and* (Y,κ) *are spaces. If A is μ-β-Lindelöf, then* f(A) *is κ-β-Lindelöf.*

Proof Suppose that $S=\left\{S_{\alpha }:\alpha \in \Lambda \right\}$ is a cover of f(A) by *κ*-*β*-open sets. Then $A=\left\{f^{-1}\left(S_{\alpha }\right):\alpha \in \Lambda \right\}$ is a cover of *A*, but *f* is $\left(\omega_{\beta_{\mu }},\beta_{\kappa }\right)$-continuous, so $f^{-1}\left(S_{\alpha }\right)$ is $\omega_{\mu }$-*β*-open for each α∈Λ. Since *A* is *μ*-*β*-Lindelöf, it follows from Proposition 5.1 that there exist $\alpha_{1},\alpha_{2},\alpha_{3},...\in \Lambda$ such that $A\subset {⋃}_{i=1}^{\infty }f^{-1}\left(S_{\alpha_{i}}\right)$. Thus $f\left(A\right)\subset {⋃}_{i=1}^{\infty }f\left(f^{-1}\left(S_{\alpha_{i}}\right)\right)\subset {⋃}_{i=1}^{\infty }S_{\alpha_{i}}$. Hence, f(A) is *κ*-*β*-Lindelöf. □

Corollary 5.4 *Let* f:(X,μ)→(Y,κ) *be a* $\left(\beta_{\mu },\beta_{\kappa }\right)$ *-continuous function, where* (X,μ) *and* (Y,κ) *are spaces. If A is μ-β-Lindelöf, then* f(A) *is κ-β-Lindelöf.*

Now, we are ready to study a product property of *μ*-*β*-Lindelöf spaces; to proceed, we recall the following.

Proposition 5.5 *[12]* *Let* (X,μ) *and* (Y,κ) *be GTSs, and let* U={U×V:U∈μ,V∈κ} *. Then* λ={⋃V:V⊂U} *is a GT on* X×Y *.*

Remark 5.6 [12] Let (X,μ) and (Y,κ) be GTSs. Then *λ* from Proposition 5.5 is called the generalized product topology on X×Y, and denoted by μ×κ. Let A⊂X, B⊂Y, and K⊂X×Y. Then it is easy to see the following:

(i) $c_{\lambda }\left(A\times B\right)=c_{\mu }\left(A\right)\times c_{\kappa }\left(B\right)$;

(ii) $i_{\lambda }\left(A\times B\right)=i_{\mu }\left(A\right)\times i_{\kappa }\left(B\right)$.

Lemma 5.7 *Let* (X,μ) *and* (Y,κ) *be spaces, and λ be the generalized product topology on* X×Y *. Then the projection* $P_{X}:\left(X\times Y,\lambda \right)\to \left(X,\mu \right)$ *(resp.* $P_{Y}:\left(X\times Y,\lambda \right)\to \left(Y,\kappa \right)$ *) is* $\left(\beta_{\lambda },\beta_{\mu }\right)$ *-continuous (resp.* $\left(\beta_{\lambda },\beta_{\kappa }\right)$ *-continuous).*

Proof We will show that the projection $P_{X}:\left(X\times Y,\lambda \right)\to \left(X,\mu \right)$ is $\left(\beta_{\lambda },\beta_{\mu }\right)$-continuous, the other case is similar. Let *A* be a *μ*-*β*-open subset of *X*. Then ${\left(P_{X}\right)}^{-1}\left(A\right)=A\times Y$. We want to show that A×Y is *λ*-*β*-open. Now by Remark 5.6, $c_{\lambda }\left(i_{\lambda }\left(c_{\lambda }\left(A\times Y\right)\right)\right)=c_{\mu }\left(i_{\mu }\left(c_{\mu }\left(A\right)\right)\right)\times Y⊃A\times Y$. Thus, A×Y is *λ*-*β*-open. □

Corollary 5.8 *Let* (X,μ) *and* (Y,κ) *be spaces, and λ be the generalized product topology on* X×Y *. If* X×Y *is λ-β-Lindelöf, then* (X,μ) *is μ-β-Lindelöf and* (Y,κ) *is κ-β-Lindelöf.*

Proof Follows from Corollary 5.4 and Lemma 5.7. □

Proposition 5.9 *Let* f:(X,μ)→(Y,κ) *be a* $\left(\beta_{\mu },\omega_{\beta_{\kappa }}\right)$ *-closed function, where* (X,μ) *and* (Y,κ) *are spaces. If for each* y∈Y *,* $f^{-1}\left(y\right)$ *is μ-β-Lindelöf, then* $f^{-1}\left(A\right)$ *is μ-β-Lindelöf whenever A is κ-β-Lindelöf.*

Proof Suppose that $S=\left\{S_{\alpha }:\alpha \in \Lambda \right\}$ is a cover of $f^{-1}(A)$ by *μ*-*β*-open sets. Then it follows by assumption that for each y∈A there exists a countable subcollection $S^{y}$ of S such that $f^{-1}\left(y\right)\subset ⋃S^{y}$. Let $V_{y}=⋃S^{y}$. Then $V_{y}$ is *μ*-*β*-open. Let $H_{y}=Y﹨f\left(X﹨V_{y}\right)$. Then $H_{y}$ is $\omega_{\kappa }$-*β*-open as *f* is $\left(\beta_{\mu },\omega_{\beta_{\kappa }}\right)$-closed, also $y\in H_{y}$ for each y∈A as $f^{-1}\left(y\right)\subset V_{y}$. Thus, $H=\left\{H_{y}:y\in A\right\}$ is a cover of *A* by $\omega_{\kappa }$-*β*-open sets, but *A* is *κ*-*β*-Lindelöf, so it follows from Proposition 5.1 that there exist $y_{1},y_{2},y_{3},...\in A$ such that $A\subset {⋃}_{i=1}^{\infty }H_{y_{i}}$. Thus, $f^{-1}\left(A\right)\subset {⋃}_{i=1}^{\infty }f^{-1}\left(H_{y_{i}}\right)\subset {⋃}_{i=1}^{\infty }V_{y_{i}}$. Since $S^{y_{i}}$ is a countable subcollection of S for each i∈N, it follows that ${⋃}_{i=1}^{\infty }S^{y_{i}}$ is a countable subcollection of S. Hence, $f^{-1}\left(A\right)$ is *μ*-*β*-Lindelöf. □

Corollary 5.10 *Let* f:(X,μ)→(Y,κ) *be a* $\left(\beta_{\mu },\beta_{\kappa }\right)$ *-closed function, where* (X,μ) *and* (Y,κ) *are spaces. If for each* y∈Y *,* $f^{-1}\left(y\right)$ *is μ-β-Lindelöf, then* $f^{-1}\left(A\right)$ *is μ-β-Lindelöf whenever A is κ-β-Lindelöf.*

## Funding statement

This research did not receive any specific grant from funding agencies in the public, commercial, or not-for-profit sectors.

## CRediT authorship contribution statement

Mohammad Shawqi Sarsak: Conceived and designed the experiments; Performed the experiments; Analyzed and interpreted the data; Contributed reagents, materials, analysis tools or data; Wrote the paper.

## Declaration of Competing Interest

The authors declare no conflict of interest.

## Data Availability

No data was used for the research described in the article.
